# Deep Neural Network Model of Hearing-Impaired Speech-in-Noise Perception

**DOI:** 10.3389/fnins.2020.588448

**Published:** 2020-12-15

**Authors:** Stephanie Haro, Christopher J. Smalt, Gregory A. Ciccarelli, Thomas F. Quatieri

**Affiliations:** ^1^Human Health and Performance Systems, Massachusetts Institute of Technology Lincoln Laboratory, Lexington, MA, United States; ^2^Speech and Hearing Biosciences and Technology, Harvard Medical School, Boston, MA, United States

**Keywords:** speech-in-noise (SIN), deep neural network (DNN), cochlear modeling, cochlear synapatopathy, medial olivocochlear (MOC) efferents

## Abstract

Many individuals struggle to understand speech in listening scenarios that include reverberation and background noise. An individual's ability to understand speech arises from a combination of peripheral auditory function, central auditory function, and general cognitive abilities. The interaction of these factors complicates the prescription of treatment or therapy to improve hearing function. Damage to the auditory periphery can be studied in animals; however, this method alone is not enough to understand the impact of hearing loss on speech perception. Computational auditory models bridge the gap between animal studies and human speech perception. Perturbations to the modeled auditory systems can permit mechanism-based investigations into observed human behavior. In this study, we propose a computational model that accounts for the complex interactions between different hearing damage mechanisms and simulates human speech-in-noise perception. The model performs a digit classification task as a human would, with only acoustic sound pressure as input. Thus, we can use the model's performance as a proxy for human performance. This two-stage model consists of a biophysical cochlear-nerve spike generator followed by a deep neural network (DNN) classifier. We hypothesize that sudden damage to the periphery affects speech perception and that central nervous system adaptation over time may compensate for peripheral hearing damage. Our model achieved human-like performance across signal-to-noise ratios (SNRs) under normal-hearing (NH) cochlear settings, achieving 50% digit recognition accuracy at −20.7 dB SNR. Results were comparable to eight NH participants on the same task who achieved 50% behavioral performance at −22 dB SNR. We also simulated medial olivocochlear reflex (MOCR) and auditory nerve fiber (ANF) loss, which worsened digit-recognition accuracy at lower SNRs compared to higher SNRs. Our simulated performance following ANF loss is consistent with the hypothesis that cochlear synaptopathy impacts communication in background noise more so than in quiet. Following the insult of various cochlear degradations, we implemented extreme and conservative adaptation through the DNN. At the lowest SNRs (<0 dB), both adapted models were unable to fully recover NH performance, even with hundreds of thousands of training samples. This implies a limit on performance recovery following peripheral damage in our human-inspired DNN architecture.

## 1. Introduction

It is a universal human experience that background noise and reverberation make it harder to understand speech, and this phenomenon is exacerbated for those who suffer from hearing loss. Even individuals with normal clinical hearing tests (i.e., pure-tone audiograms) can have difficulty understanding speech in noise (Liberman et al., 2016). Deficits in an individual's speech perception may impact their quality of life, including physical, financial, social and emotional dimensions (Ciorba et al., 2012). The degree to which noise impacts an individual's ability to discern spoken words (i.e., their speech perception) varies, and difficulties in these scenarios arise from a combination of deficits in peripheral auditory function, central auditory function, and general cognitive abilities (Frisina and Frisina, 1997; Plack et al., 2014; Heinrich et al., 2015; Parthasarathy et al., 2020). Unfortunately, directly assessing the impact of peripheral or central factors on speech perception would require invasive human studies that are typically not possible.

While isolating the cause of speech perception in human studies can be challenging, over the past decade animal studies have shown that noise exposures cause a permanent loss of low-spontaneous-rate auditory nerve fibers (ANFs) and reduction of auditory brainstem response (ABR) wave-I amplitudes (Kujawa and Liberman, 2009). This phenomenon is termed cochlear synaptopathy, and is thought to create difficulties understanding speech in noise for humans, while not producing changes that are reflected in the clinical pure-tone-threshold audiogram. There is currently no established technique to measure cochlear synaptopathy non-invasively in humans. As a result, it is difficult to translate noise-induced hearing loss findings from animal studies into perceptual measures of human auditory function (Plack et al., 2014; Bramhall et al., 2019; Le Prell et al., 2019). Unlike pure-tone audiometric testing, where threshold shifts can be converted into cochlear inner hair cell (IHC) and outer hair cell (OHC) health estimates, a speech-in-noise assessment score currently does not have physiological interpretation. Oxenham (2016) predicted that a 50% loss of low-spontaneous-rate ANFs would cause a negligible decline in performance on psychoacoustic tasks, including tone detection in noise. This information theory approach, however, did not directly assess speech perception.

Computational auditory models provide a bridge between studying the effects of controlled noise exposures in animals and estimating the perceptual outcomes of humans in real-world environments (Tepe et al., 2017; Verhulst et al., 2018; Le Prell et al., 2019). Recent versions of the auditory periphery model (Zilany et al., 2014) potentially provide a more accurate representation of human cochlear tuning and can be adjusted to match the audiogram of an individual person. Furthermore, human cochlear models also have the capacity to simulate cochlear synaptopathy which is hypothesized to contribute to human speech-in-noise perception difficulties (Bharadwaj et al., 2014; Bruce et al., 2015; Smalt et al., 2016; Keshishzadeh and Verhulst, 2019).

Many existing speech intelligibility metrics rely on much simpler cochlear models, and therefore they are limited in their capacity to predict human speech perception performance. For example, the speech transmission index (STI), a commonly used measure of intelligibility, outputs a normalized prediction between 0 and 1. However, STI was not designed to take into account cochlear non-linearities (such as the effect of overall sound level) and hearing loss (Houtgast et al., 1980; Taal et al., 2011). Elhilali et al. (2003) developed an extension of the STI, referred to as the spectro-temporal modulation index (STMI) to account for cochlear non-linearities and account effect phase shifts in the acoustic waveform, but their method does not address the impact of hearing loss.

To account for hearing loss, speech-in-noise intelligibility metrics can be estimated from the neurogram, a simulated auditory nerve population's response produced by phenomenological cochlear modeling. Zilany and Bruce (2007) used the neurogram as input to the STMI, allowing the intelligibility prediction to account for hair-cell damage. Hines and Harte (2012) derived an alternative metric called the Neurogram Similarity Metric (NSIM), which directly compares a clean and degraded neurogram model output and is less computationally intensive.

Another disadvantage shared by many current speech intelligibility prediction metrics is the need for a clean reference speech waveform that is separated from the noise or degradation. Such metrics ultimately rely on quantifying degradation in the signal's acoustic properties and do not directly capture physiological components of perception. The Bispectrum (Hossain et al., 2016, 2019) is an alternative reference-free technique that is similar in terms of computational complexity, but the mapping between the metric and perceptual performance may need to be tuned for a specific speech-in-noise task. In addition, the Bispectrum does not capture the potential effects of semantics or vocabulary. In general, the classic speech intelligibility metrics lack the ability to account for the contribution of the auditory cortex and other parts of the brain that are involved in speech recognition, and so they cannot be directly used to assess perceptual performance.

An alternative approach to estimating speech intelligibility is to couple auditory-periphery inspired signal processing to a speech recognition system that performs a stimulus-in-noise classification task. The performance of the recognition system can be directly related to human performance without a clean reference signal for comparison. Several studies have used a deep neural network (DNN) based automatic speech recognition (ASR) system with a periphery-inspired front-end to simulate speech intelligibility under various conditions (Moritz et al., 2015; Kell et al., 2018; Spille et al., 2018; Arai et al., 2019). There are a few studies that also extend their model to study the impact of audiometric losses on their simulated performance (Fontan et al., 2017; Schilling et al., 2020). However, it is hypothesized that individuals with normal audiometric profiles may still have issues with speech intelligibility due to cochlear synaptopathy (Liberman et al., 2016). A series of studies which used a cochlear model paired with a non-DNN speech recognizer found a simulated speech intelligibility performance improvement when the medial olivocochlear reflex (MOCR) was incorporated into the model (Brown et al., 2010; Clark et al., 2012). The MOCR efferent feedback reduces OHC gain, providing an anti-masking effect in background noise, resulting in improved digit recognition accuracy (Backus and Guinan, 2006).

**Figure 1:**
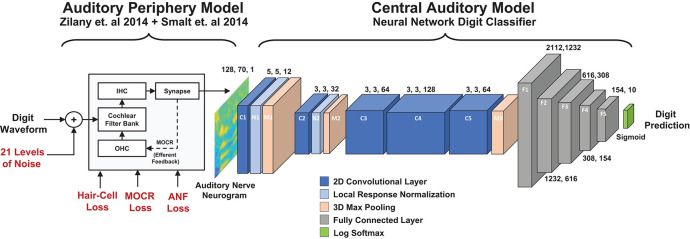
Diagram of the two stage digit-in-noise processing model. This two-stage model takes an acoustic waveform as its input and outputs a prediction of the spoken digit in that waveform. For the first stage, the peripheral model of Smalt et al. (2014) and Zilany et al. (2014) has the ability to implement audiometric, medial olivocochlear reflex (MOCR) and auditory nerve fiber (ANF) losses and produces auditory neurograms. For computational reasons, we down sampled and z-score normalized this output and use it as the input to the digit-in-noise classification network. The second stage of the model, a DNN digit classifier is constituted by convolutional and fully connected layers that have normalization and max pooling layers interspersed and was inspired by the architecture of Kell 2018 for its relevance to human neurobiology.

This study's aim was to develop an end-to-end model of the auditory system that uses a biophysical cochlear front-end with a DNN representation of the brainstem, midbrain, and cortex (Figure 1). We used this model to study the effects of audiometric, MOCR, and ANF cochlear degradations on speech perception performance. This work is novel relative to other biologically-inspired ASR systems since it explored a combination of cochlear degradation types as well as subsequent central adaption. In the first stage, we used the cochlear periphery model to generate neurogram responses to spoken digits presented in background noise with a variety of hearing loss configurations. In the second stage, we then classified the neurograms using a DNN. We used this resulting model to perform a closed-set, digit-recognition-in-noise task across a range of SNRs, and produced a digit recognition accuracy curve that can be compared against human speech-in-noise psychometric curves. We explored three main goals in this work. The first was to test if using a spiking cochlear model (instead of acoustic waveforms) could be used to replicate human-like speech recognition. Our second goal was to test how various cochlear model degradations affect the performance of the digit-in-noise classifier. Third, we compared how two simulations of neural plasticity affected the DNN classifier's ability to adapt to the cochlear degradations. As part of this last aim we proposed more conservative bounds around how much hearing performance recovery is possible following retraining.

## 2. Methods

### 2.1. Pure-Tone Audiometric Characterization of Subjects

**Figure 2:**
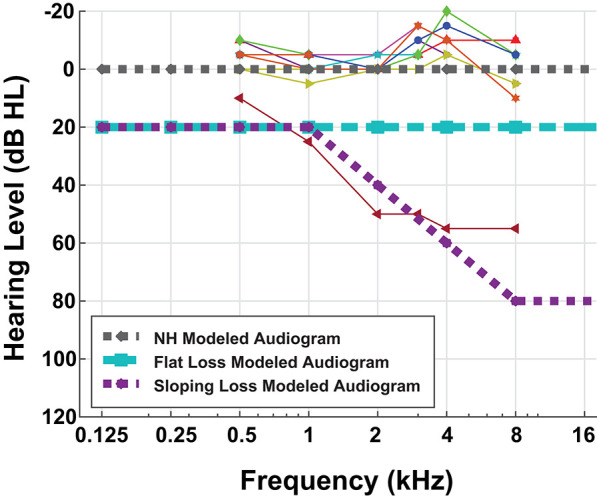
Behavioral and modeled pure-tone audiograms. This figure overlays the individual participant left ear audiogram results (thin lines) with the three modeled audiometric profiles (thick dashed lines). Eight out of nine of our participants have normal-hearing (NH) audiograms, i.e., flat pure-tone thresholds near 0 dB hearing level. One of our participants had characteristic sloping hearing loss so we excluded them from further analysis. Although pure-tone thresholds do not consistently correspond with speech difficulties, we performed this hearing assessment for completeness.

Nine MIT Lincoln Laboratory employees (seven male, two female) gave written informed consent to participate in a pure-tone audiometric hearing test in addition to a digit-in-noise assessment. The MIT Committee on the Use of Humans as Experimental Subjects and the US Army Medical Research and Materiel Command (USAMRMC) Human Research Protection Office approved the experimental protocol. We conducted all research in accordance with the relevant guidelines and regulations for human-subject testing required by these committees. Since our two-stage model is representative of the framework of one ear, we are reporting the assessment results for each participant's left ear only. We administered the audiogram using Wireless Automated Hearing Test System (WAHTS) headphones (Creare LLC., Hanover NH) in a sound-treated booth. The automated audiogram evaluates pure-tone thresholds at [0.125, 0.250, 0.5, 1, 2, 4, 8] kHz. Figure 2 contains behavioral audiometry for each of the nine participants. Figure 2 also shows the three modeled audiometric profiles superimposed with participant left ear audiograms. Eight participants self-reported normal hearing and subsequently produced normal-hearing (NH), non-shifted flat pure tone audiometric results around 0 dB Hearing Level (HL). One hearing-impaired participant stands out from the cohort due to their exhibited high-frequency sloping hearing loss. We did not perform further analysis on this subject.

### 2.2. Digit-in-Noise Task

To evaluate speech perception performance, we used a digit-in-noise speech identification task. This task was performed by both human participants and our computational model. Human participants listened to the the spoken digit in noise, and were asked to identify that digit. The computational model was given a digital representation of underlying acoustic waveforms, with the output being a prediction of the spoken digit in that waveform. Our digit-in-noise assessment used the TIDIGITS, a set of acoustic recordings of spoken digits (Leonard and Doddington, 1993). Our digit-in-noise assessment used the shorter, single-digit utterances zero through nine, instead of the conventionally used triple-digit utterances because processing stimuli through the auditory periphery model is extremely computationally expensive. Each of the dataset's 225 talkers (111 male and 114 female) contained two recorded single-digit utterances, resulting in 4,500 unique single-digit utterances. We set each of the unique utterances to a stimulus level of 70 dB SPL and digitally added flat-spectrum, white noise between [0, 100] dB SPL at 5 dB steps, amounting to 94,500 waveforms. Each of these waveforms were individually processed through the auditory periphery model that we describe in detail in section 2.3.1.

**Figure 3:**
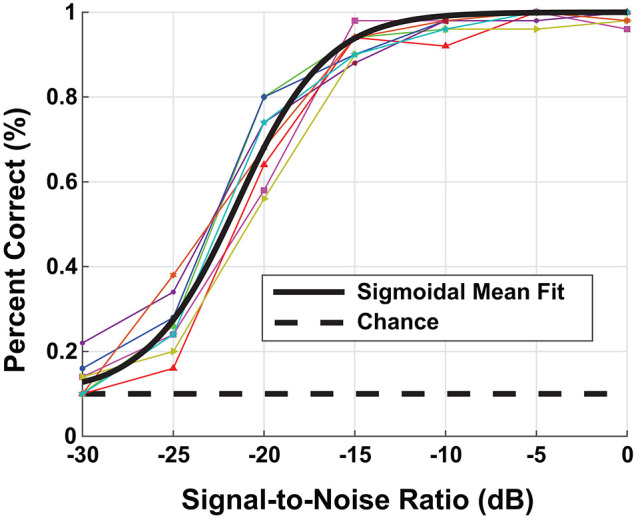
Behavioral digit-in-noise assessment. The colored lines represent the individual participant digit-in-noise performance curves and the black curve is a sigmoidal fit applied to the mean digit-in-noise performance taken across normal-hearing (NH) participants. We use this NH behavioral curve as the benchmark digit recognition accuracy curve when optimizing the DNN.

Using a random sampling of the TIDIGIT waveform data base, we ran a speech-in-noise test on the the eight participants with NH, i.e., non-shifted, flat-pure-tone audiograms. The human assessment consisted of 100 individual trials at each background-noise level presented to the left ear through Sennheiser HD598 headphones. We presented the speech stimulus at a fixed sound level of 65 dB SPL and varied the noise level to produce test stimuli with SNRs between [−30, 0] dB. Figure 3 contains the digit-in-noise performance for each of the eight NH participants. Using the following equation, we computed a sigmoid fit on the mean speech perception performance curve computed across the NH subset of participants:

(1) $$\begin{array}{r} f_{\text{human}}\left(x\right)=0.1+\frac{0.9}{\left(1+exp\left(-a\left(x-b\right)\right)\right)}. \end{array}$$

This sigmoid fit is plotted in black in Figure 3. We used MATLAB's (Mathworks, Natick, MA) fit function to solve for parameters *a* and *b* using non-linear least squares. To provide an accurate performance benchmark for the full model described in Figure 1, we ran the digit-in-noise assessment on self-reported, NH participants. **Figures 6, 8** each have the mean participant performance curve overlaid on each panel to serve as an unimpaired human reference for the model-based predictions in the sections that follow.

### 2.3. Computational Model

#### 2.3.1. Auditory Nerve Model

To simulate ANF synapse output in response to digit-in-noise utterances, we utilized the cat auditory nerve model by Zilany (Zilany and Bruce, 2006; Zilany et al., 2009) and the more recent ‘humanized’ version (Zilany et al., 2014). Various groups have thoroughly compared this model with physiological responses to a variety of stimuli including tones, speech, and noise (Carney, 1993; Heinz et al., 2001; Zhang et al., 2001; Tan and Carney, 2003, 2005; Zilany and Bruce, 2006, 2007). This model implementation uses time-varying non-linear filters that predict physiological responses from the cat auditory system, including compression, suppression, and broadened tuning. The model uses two parameters to control IHC and OHC loss. We created three pure-tone threshold profiles representing the following: a NH, non-shifted flat audiogram; an audiogram reflective of a constant flat-threshold shift across all frequencies; and lastly, a high-frequency threshold shift referred to as sloping loss. The MATLAB fitaudiogram2 method from Zilany et al. (2009) takes the audiometric profiles as input to estimate the amount of IHC and OHC loss assuming a 1/3 and 2/3 ratio of loss between IHCs and IHCs (Zilany and Bruce, 2007; Zilany et al., 2009). Figure 2 contains the three pure-tone profiles plotted as the three thick dashed lines. For each of the three audiometric profiles, we applied two ANF and two MOCR degradation conditions for a total of 12 modeled peripheral states (Table 1). The peripheral model takes in an MOCR gain parameter that is either healthy (20 dB) or degraded (0 dB). We modeled 100 ANFs per cochlear frequency band and partitioned each set of 100 fibers into 20 low, 20 medium, and 60 high spontaneous-rate ANFs, which matches the physiologically-observed distributions in the cat (Liberman, 1978). However, the total number of central frequencies (hair cells) and ANFs in this model is less than the human cochlea, due to computational limits. Although Carney (2018) proposed that high spontaneous rate ANFs are crucial for speech, we decided to model primarily low and medium spontaneous rate ANF loss per the rationale etched out by Furman et al. (2013). Additionally, we chose to model the extremes of ANF functionality, i.e., a healthy distribution ([20, 20, 60]) and a degraded distribution ([0, 0, 52]) in our proof of concept model, even though Kujawa and Liberman (2009) reports more conservative 50% ANF loss following noise exposure.

**Table 1:** Combination of hearing loss used in model simulations.

**Audiometric profile**	**MOCR gain (dB)**	**Auditory nerve fiber types ([low, medium, high])**
NH	20	[20, 20, 60]
NH	0	[20, 20, 60]
NH	20	[0, 0, 52]
NH	0	[0, 0, 52]
Flat loss	20	[20, 20, 60]
Flat loss	0	[20, 20, 60]
Flat loss	20	[0, 0, 52]
Flat loss	0	[0, 0, 52]
Sloping loss	20	[20, 20, 60]
Sloping loss	0	[20, 20, 60]
Sloping loss	20	[0, 0, 52]
Sloping loss	0	[0, 0, 52]

*The 12 cochlear states studied in this paper are a result of four combinations of MOCR and ANF degradations applied to each of the three modeled audiometric profiles*.

We combined the auditory model of Zilany et al. (2014) with a model of the MOCR of Smalt et al. (2014) that can simulate a time and frequency dependent anti-masking effect thought to be important for speech-in-noise perception (Brown et al., 2010; Chintanpalli et al., 2012; Clark et al., 2012). The MOCR in the model adapts the gain of the OHC (*cohc*) based on the OHC pathway input of the model. To adapt this model for human use, we shifted the frequency band sensitive to MOCR effects down to the human range using the Greenwood function (Greenwood, 1961). The strength of the reflex can be manipulated by the parameter *MOCR*_*Max*_, and can range from 0 to 1, where 1 represents the maximum gain reduction possible and is equal to the OHC gain available at that center frequency (CF).

To simulate neural responses to speech-in-noise stimuli, we ran the auditory nerve model at CFs ranging from [100 Hz, 8 kHz] in 128 logarithmic spaced steps. At each CF we simulated 100 ANF spiking responses. This stimuli representation provides both narrow band and wide band frequency resolution required to resolve harmonic and formant information. Similar to Zilany and Bruce (2007), we added up the spike response at each CF, and summed the energy in time with a non-overlapping 8 ms window to produce a neurogram representation of the stimuli. We downsampled the neurograms from 100 kHz to 100 Hz to reduce the dimensions to a manageable size for the DNN. Then, we z-score normalized each of the neurograms independently before they were used for training and testing the DNN stage of the model. We discuss our rationale for z-scoring, in detail, in section 2.3.3.

**Figure 4:**
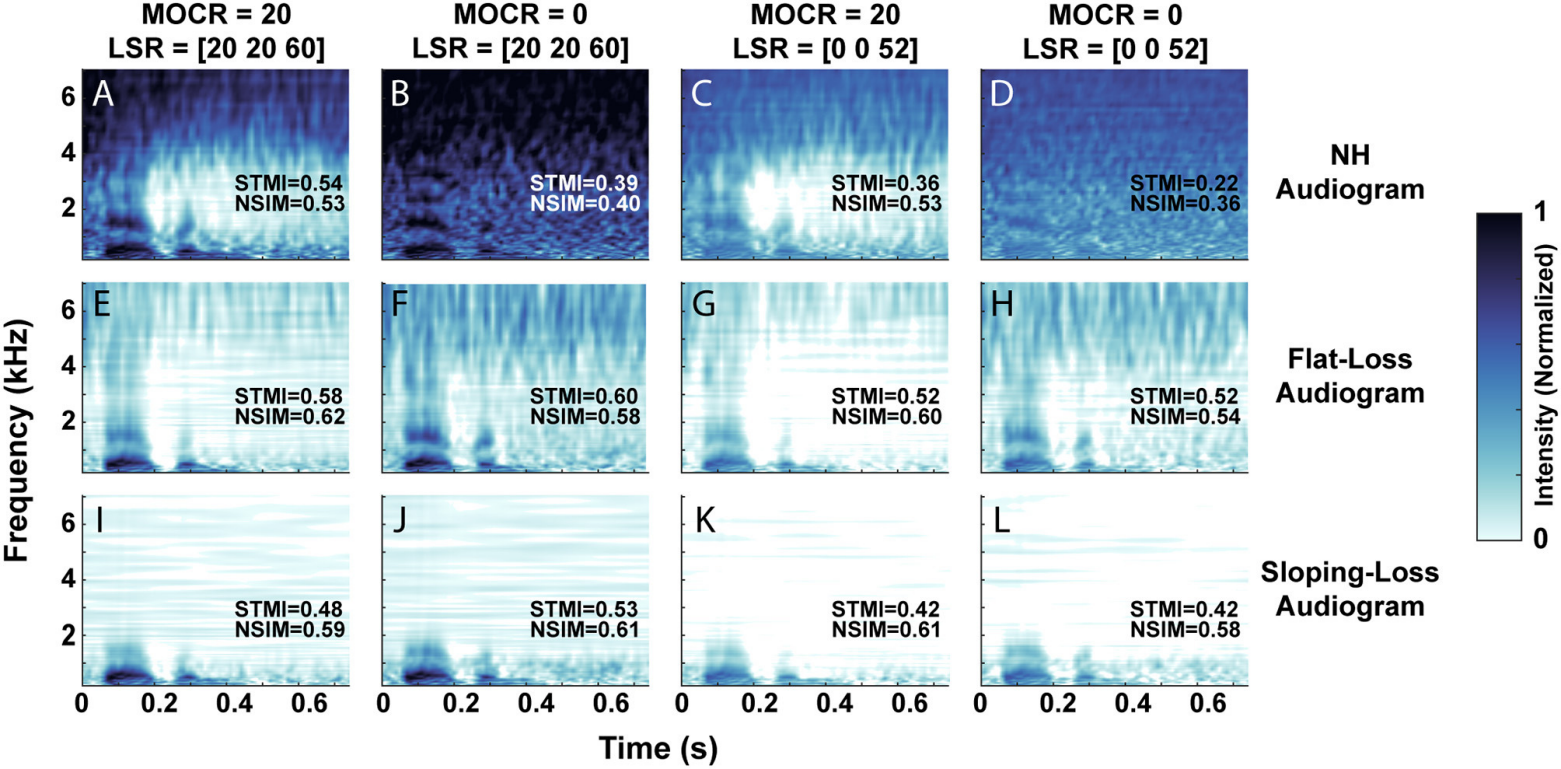
Neurogram representation of the digit ‘seven’ in 5 dB SNR of white background noise. **(A–L)** Illustrates the 12 cochlear model states, organized by their audiometric profiles (rows) and combination of medial olivocochlear reflex (MOCR) and auditory nerve fiber (ANF) loss (columns). We modeled an MOCR gain of either 0 or 20 dB and a distribution of either [20, 20, 60] or [0, 0, 52] low, medium, and high spontaneous rate auditory nerve fibers. **(A)** Represents the utterance processed with the normal-hearing (NH) cochlear setting, while **(L)** illustrates the utterance processed through the most degraded cochlear state studied. Each image has objective reference-based predictions of intelligibility (STMI and NSIM) which indicate a loss of intelligibility as simulated cochlear damage increases (a 0-1 scale, where 1 represents 100% intelligibility).

Figure 4 visualizes the neurogram representation of an utterance after we processed it through 12 combinations of audiometric, MOCR, and ANF degradations. We presented a single talker's utterance of the digit ‘seven’ in 5 dB SNR of white background noise. Each row represents the neurogram output using the three modeled audiometric profiles, while the columns iterate through the four ANF and MOCR model settings. Figure 4A represents NH cochlear function, illustrating the spectral and temporal resolution that the model provides with no degradation present. A comparison of the first and second columns reveals the anti-masking effects of the MOCR, which occurs between ~1 and 4 kHz. An overall lower signal level and reduced clarity of the formants can be observed between the first and third columns due to the loss in auditory-nerve fibers. It takes on the the order of 20 min on a single CPU core to generate a neurogram for a single utterance for a given cochlear state. Using the the MIT Lincoln Laboratory Supercomputing Center (LLSC), we generated 94,500 unique neurograms on ~8,192 cores using parallelization implemented in pMatlab (Reuther et al., 2018).

This set of neurograms can be used to illustrate a drawback of standard metrics of speech intelligibility perception, such as STMI and NSIM. We computed the objective STMI (Elhilali et al., 2003) and NSIM (Hines and Harte, 2012) metrics for each neurogram. Although these measures range from 0 to 1, with 1 representing perfect intelligibility as compared to a NH reference neurogram in the absence of background noise, they can not directly be compared to behavioral task accuracies. In some cases the objective metrics indicate that the neurograms with audiometric loss are in fact more intelligible than the NH pure-tone neurogram. For example, in Figure 4E, both STMI and NSIM indicate that the flat-loss audiogram representation is more intelligible than the NH audiogram representation. The modeled flat-loss is reducing the amount of signal at the auditory nerve of both the noise and digit stimulus, so the STMI and NSIM report less degradation in the flat-loss audiometric case than in the NH-audiometric case. However, in the context of speech intelligibility, an audiometrically degraded stimulus should not have a better predicted speech intelligibility score. This inconsistency motivates the need for a more comprehensive intelligibility metric that is biologically inspired and factors in additional central processing.

#### 2.3.2. Digit Classifier DNN Architecture

Figure 1 illustrates an overview of our model of speech perception that is composed of a biophysical auditory periphery and a DNN representation of central processing. Unlike conventional DNN-based ASR systems that operate on speech waveforms or spectrograms, this DNN used the cochlear neurogram as its input data. We utilized a neural network classifier to model human post-cochlear neural processing to perform a digit-in-noise task. The DNN served as an optimal observer whose goal was to train itself to maximize task performance (Geisler, 2011), no matter the type of cochlear degradation. To clarify, we did not train the DNN to make the same decisions (identification mistakes) found in human perception. Instead one of our goals was to see if the classifier learned similar behavior on its own. Furthermore, we know of no human data set of speech perception confusion matrices that are paired with ANF and MOCR measures, so this study explores potential upper and lower bounds for performance in these scenarios. Since the DNN required inputs of the the same spectrotemporal dimensions, we trimmed all neurograms to have the same 700 ms length from their onset. Additionally, we z-score normalized the neurogram outputs independently of each other. This processing step is explained in detail in section 2.3.3 after our train/test paradigm framework has been thoroughly presented. We used PyTorch version 1.3 to implement all processes relating to the DNN model (Paszke et al., 2019).

Figure 1 illustrates the structural hyperparameters chosen for the DNN architecture, including layer types and dimensions. These network hyperparameters are distinct from network parameters that the network solves for, such as weights and biases. Network hyperparameters indicate the operations the network should contain in its architecture layers. The network consisted of a set of convolutional layers followed by a set of fully connected layers. Convolutional layers were used since they would preserve the two dimensional stimuli processing seen in spectrotemporal receptive fields in the auditory cortex (David et al., 2009; Schönwiesner and Zatorre, 2009). Additionally, given the two dimensional similarity between our spectrotemporal representation of stimuli and images, it was promising to use convolutional layers that have found success in DNN-based image analysis. There exist other ASR models (Kell et al., 2018; Schilling et al., 2020) and models of the auditory cortex (Akbari et al., 2019; Rahman et al., 2019) that use convolutional layer-based DNN models. We selected Kell et al. (2018)'s model because of its task similarity and validation against human fMRI imaging data of the auditory cortex.

Our convolutional layer architecture (C1 through C5) is a modified version of the convolutional layer architecture found in Kell et al. (2018)'s DNN model. The exact network hyperparameters differ due to the difference in input stimulus dimensions and number of output categories, however we preserved the ratio of layer dimensions. Our DNN includes five convolutional layers whose layer channel depths dilate and constrict by a factor of two between neighboring layers. The first set of convolutional weights are 5 × 5 pixels in dimension, while the subsequent convolutional dimensions are 3 × 3 pixels. Local-response normalization and max-pooling layers follow the first two convolutional layers. Another max-pooling layer follows the five convolutional layers before a set of five fully connected layers which gradually decrease in size. The local-response step normalizes adjacent convolutional layer output channel values while the max-pooling step provides dimensionality reduction by finding local maxima around a specified sliding window. DNNs widely use normalization (such as z-score and local-response normalization) and local maxima pooling due to their neural plausibility (Serre et al., 2007; Carandini and Heeger, 2012). We did not perform additional layer regularization beyond z-scoring the neurogram input and the two local response normalization layers. Fully connected layers are incorporated into the model because they are modeled after the fundamental units of computation in the brain (London and Häusser, 2005).

The following other hyperparameters define how the network learns its weight parameters. The network uses a batch size of 256 neurograms for each pass through the network during training. One epoch is defined as a pass of the entire set of training examples through the network before they are used again for more precise parameter value updates. Each epoch in our training contains 66,150 unique training neurograms. The number of epochs used in training will be discussed later. We used the Adam optimizer (Kingma and Ba, 2014) with an equal learning rate and weight decay of 1e-3. The network utilizes PyTorch's cross-entropy loss function which is composed of a log softmax activation function followed by a negative log likelihood loss function (Paszke, 2019). For each batch of neurograms trained through the network, loss is computed between the target class and the predicted class. This cross-entropy loss function is used when training a DNN for a classification task as opposed to a regression task.

A trained DNN's digit recognition accuracy is variable and depends on factors, such as the exact training data that is presented and the order in which the training samples are used to update the weights. To mitigate the effects of these variables in biasing our conclusions, we repeated the training and testing process for each DNN model 10 times. Each fold contained a random split of all the 94,500 unique neurograms in the form of a training, validation, and testing subset. All SNRs are sampled in each of the subsets. For every DNN model, we trained and evaluated the model on 10 different partitions (folds) of the relevant neurogram type. As a percentage of the available data set, the training data consisted of 70% of the available data, and the validation and testing data consisted of 10 and 20%, respectively following accepted ranges in the literature. We used Scikit-learn's random permutation cross-validator to create these non-identical data splits (Pedregosa et al., 2011). Consequently, the final trained weights of each of the 10-folds were slightly different. All of the visualized model digit recognition accuracy curves are composed of the mean with standard error of the mean computed across these 10-folds. Quantifying variability of digit recognition accuracy within a given train/test paradigm provides context for interpreting variability of digit recognition accuracy across paradigms.

#### 2.3.3. Implementing Adaptation-Inspired DNN Train/Test Paradigms

**Figure 5:**
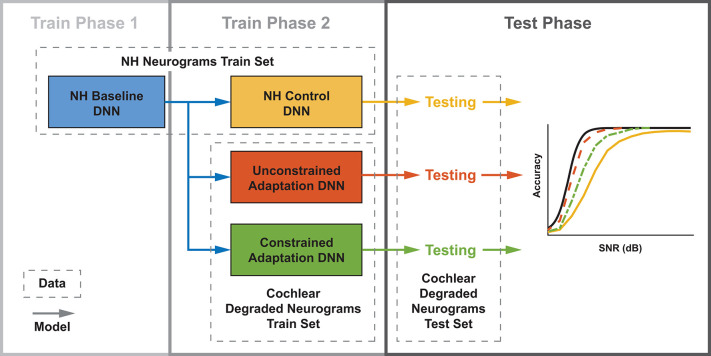
Three DNN training paradigms used to study adaptation following cochlear degradation. This training and testing framework is run for each of the 12 cochlear states that we modeled. Both training and testing data sets contain neurograms from all 21 SNRs. Normal-hearing (NH) neurograms are used to train a baseline model, which simulates NH speech-in-noise word recognition. This baseline network is utilized by three networks during a second phase of training. During phase 2, the NH control is trained on NH neurograms and the unconstrained and constrained adaptation paradigms are trained on degraded neurograms. All three of the resulting models are then tested on a held out set of degraded neurograms. The end result is digit recognition accuracy as a function of SNR, which simulates a psychometric function. The NH-control paradigm (yellow) simulates performance after a sudden hearing loss, the unconstrained-adaptation paradigm simulates unlimited training after hearing loss (red-orange), and the constrained-adaptation paradigm (green) simulates limited training following hearing loss.

We developed three training paradigms to simulate sudden cochlear damage followed by central adaptation. An overview of the training procedure is illustrated in Figure 5. The first phase of each training paradigm is identical: a baseline model was trained using only NH neurograms. This NH-baseline model simulates a human who is born with normal hearing. In the second phase of training, we further trained the NH-baseline model with additional neurograms under three different conditions. The first condition is the NH-control. For the NH-control, the NH-baseline model was further trained using additional NH neurograms (illustrated in yellow in Figure 5). In the remaining two conditions, the NH-baseline model was further trained using degraded neurograms, simulating a person who started with normal hearing, then acquired hearing loss. In the first degraded condition, the DNN was allowed to adapt to the sudden hearing loss without constraint (illustrated in red-orange in Figure 5). In the second degraded condition, the DNN adaptation to sudden hearing loss was constrained; the model was only permitted to adapt in the final layer (illustrated in green in Figure 5). Our purpose in constraining the DNN's adaptation to hearing loss was to better approximate the adaptation capability of the human brain. The specific hearing loss combinations used in the second phase of model training are outlined in **Table 1**. Finally, we evaluated each of the resulting 36 models from our training procedure on additional held out neurograms with the same type of modeled cochlear settings.

In the first training phase, we trained the DNN using only NH neurograms as our baseline. We initialized the model parameters with values randomly sampled from a normal distribution. To ensure neurogram data were split in an identical fashion among the training and test sets, and balanced with regard to talker and gender, we used a fixed random seed for each of the ten cross-validation folds. Preserving the train/test splits between training phases was critical to eliminate the chance that talkers used in training could accidentally be included during testing. To set the model training duration for the first training phase, we empirically derived the minimum amount of data required to achieve peak digit recovery accuracy in the NH-baseline model. In the second training phase, we trained the DNN using the equivalent amount of data established in the NH-baseline model. This amounted to 250,000 neurograms per training phase (or ~3.8 epochs, i.e., exposure to the entire unique training set 3.8 times).

As a general rule, a DNN should be trained and tested on data sets that are independently and identically distributed, i.e., data samples must be non-overlapping but share the same statistical make-up. If there is a mismatch in train and test statistics, any machine learning technique, including DNNs, would likely show a drop in performance. The cochlear degradations we modeled reduce the spiking output; this in turn reduces the neurogram signal strength relative to the NH condition. Therefore, we expect a mismatch between the statistical distributions of the sets of NH and degraded neurograms. To account for this expected statistical mismatch, we independently z-score normalized each neurogram before the first layer of the DNN. This z-score does not reverse the loss in information due to our modeled cochlear degradations because our peripheral model is non-linear (e.g., even as a function of acoustic stimulus level). Additionally, normalization in the auditory pathway may be neurologically plausible given the widespread evidence of normalization that takes place within the cortex and specifically in primary auditory cortex (Carandini and Heeger, 2012).

To estimate neural plasticity following cochlear degradation, we varied the number of DNN parameters it was possible to change in the phase 2 training. In the unconstrained-adaptation paradigm, we permitted all parameters in every network layer to be updated. For the constrained-adaptation paradigm, we only allowed the fifth fully connected layer (F5) weights and bias to adapt during phase two of training. Finally, the NH-control training paradigm acts a static response to cochlear degradation, no adaptation is possible. In this fashion, the NH-control paradigm acts as a lower bound for neural plasticity, while the unconstrained-adaptation paradigm acts as an upper bound.

## 3. Results

In this section, we first compare our NH-model of speech perception to NH humans on the digit-in-noise task. Next, we then use our model's output to investigate how peripheral degradation impacts digit recognition accuracy, and how performance might change after further training on degraded cochlear neurograms to simulate neural plasticity. To statistically compare the performance of our various model conditions, we calculated SNR required to achieve 50% digit recognition accuracy. Then, we also present confusion matrices between the target and predicted digit classifications at a given SNR for both human data and selected models. Finally, we characterize when, where, and how the network adapts to cochlear degradation.

### 3.1. Comparison of Normal-Hearing Human and Model Digit-in-Noise Performance

**Figure 6:**
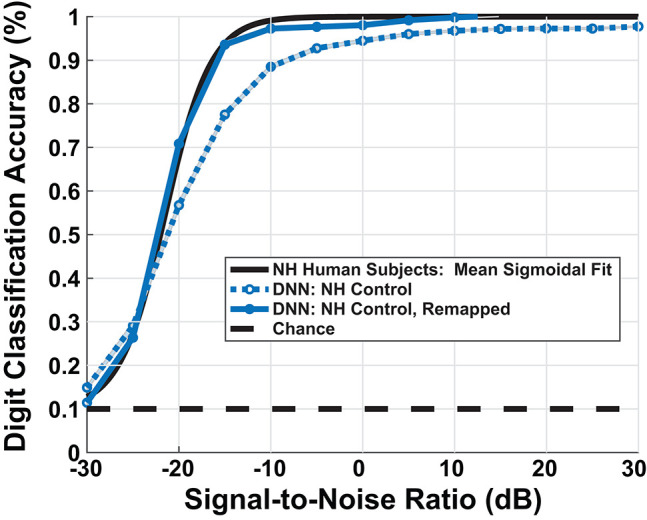
Model simulated digit-in-noise performance scaled by mean behavioral response. DNN model under the normal-hearing (NH) model produced the expected sigmoidal shape of measured human behavior (black curve), leaving a small man-machine gap of 1.3 dB SNR at 50% digit recognition accuracy. To facilitate visual, qualitative comparison between between the various models of cochlear degradation studied, we scaled the DNN output accuracies on all DNN outputs using an SNR dependent scaling factor. We computed the scaling factor used to remap all subsequent model accuracies once using the sigmoid fits of the DNN NH-model and NH human mean performance.

Our DNN model replicated the upper and lower limits of human performance over a range of SNRs, producing a sigmoidal-shaped digit recognition accuracy curve. There exists a small man-machine gap between human performance and the ‘NH-Model’ in Figure 6. Participants and the model achieved 50% accuracy at −22 and −20.7 dB SNR, respectively, indicating a gap of 1.3 dB SNR. The mean absolute accuracy in terms of percent correct performance discrepancy between human data and model between [−30, 0] dB SNR was 7.3%. Although the network achieved sigmoidal digit recognition accuracy, it did not completely replicate the mean human performance (black line), particularly at higher SNRs. We performed a sigmoid fit on the NH-model using the following equation:

(2) $$\begin{array}{r} f_{\text{model}}\left(x\right)=0.09+\frac{0.88}{\left(1+exp\left(-a\left(x-b\right)\right)\right)}. \end{array}$$

To account for a small mismatch at high SNRs between our NH hearing participants and the model, we applied a scaling factor to the estimated digit recognition accuracy. We computed the scaling factor by taking the difference between the parameters of the sigmoid fit of the mean human data and the sigmoid fit of the mean NH-model. Thus, scaling factor maps the simulated psychometric function onto a human-like performance space. This process was done so that digit recognition accuracy differences between combinations of paradigms and cochlear states could be interpreted in the same units as human behavioral performance. The dark blue curve in Figure 6 shows the impact of the scaling factor. The model's digit recognition accuracy values in **Figures 8, 10** reflect scaled results.

**Figure 7:**
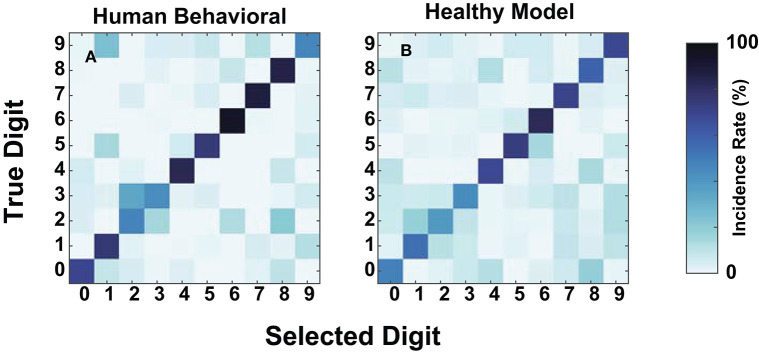
Human and normal-hearing (NH) model digit-in-noise confusions compared at −20 dB SNR. Within each panel, the rows represent the true digit and the columns convey the predicted. **(A)** Human confusion matrix. **(B)** The confusion matrix for the NH-model tested on NH neurograms.

**Figure 8:**
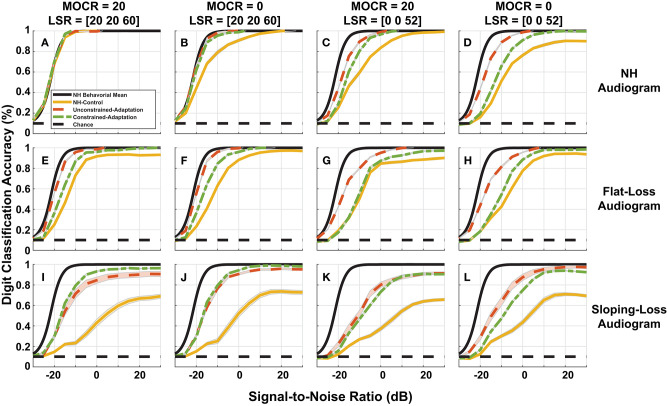
Simulated digit-in-noise performance as a function of hearing loss. **(A–L)** Illustrates the digit-in-noise accuracy for each of the three train/test paradigms. Each row corresponds to one of the three modeled audiometric profiles and the columns represent the four combinations of medial olivocochlear reflex (MOCR) and auditory nerve fiber (ANF) losses. In addition to each of the training models' digit recognition accuracy, every panel has the mean behavioral curve (black) and chance plotted to serve as a stable reference across the panels. For each cochlear state, we plot the NH-control (yellow), unconstrained-adaptation (red-orange), and constrained-adaptation (green) paradigm mean digit recognition accuracy curves with standard error.

We compared human and model confusions between digits to determine whether failure modes at these SNRs were similar. Figure 7 contains the confusions at −20 dB for the mean NH human responses and the NH-model from Figure 8A. We computed the mean human confusion matrix across the NH participants and the mean NH-model confusion matrix across 10-folds. Both the model and the human confusions produced approximately the same performance at this SNR. Their confusions look similar (Figure 7) and have a root mean squared error (RMSE) of 8.25. They both classified the digit six with the most accuracy, digits four through eight well, and more often confused digits two and three. The two and three digit confusion has been previously observed in human digit-in-noise perception (Morgan et al., 1973).

### 3.2. Effects of Cochlear Degradation and Adaptation Paradigms on Digit-in-Noise Performance

Figure 8 breaks down our performance simulations to show the impact our 12 modeled peripheral auditory degradations have on digit recognition accuracy. The 12 cochlear states shown are a combination of audiometric loss (rows) and ANF and MOCR cochlear degradation (columns). Within each of the 12 panels, we plot the DNN digit recognition accuracy for three paradigms (yellow, red-orange, and green) with the mean NH behavioral accuracy (black) from Figure 3. We illustrate a static response to the indicated combination of cochlear degradation with no opportunity to adapt when we tested the NH-control paradigm (yellow) on degraded neurograms. The unconstrained-adaptation paradigm (red-orange) serves as an upper limit of the amount of adaptation permitted in this network. The constrained-adaptation paradigm (green) is a more conservative model of plasticity following degradation since we only allowed the last layer of the network to adapt during the second phase of training. We computed the standard error for each digit recognition accuracy curve to quantify the slight variations that are due to the stochastic nature of DNN parameter training between training folds.

Figure 8A shows digit recognition accuracy across NH neurograms was nearly identical regardless of the train/test paradigm. We expected this effect because we used NH neurograms across the training phases and during testing. Given a NH audiogram, as the severity of additional modeled ANF and MOCR degradation increased (Figures 8A–D), all three of the paradigms performed more poorly relative to the NH-model (Figure 8A, yellow). This decrease in digit recognition accuracy can be interpreted as a shift toward a higher SNR required to achieve the same accuracy or a change in SNR required to achieve 50% digit recognition accuracy. The NH-control paradigm (yellow) serves as a snapshot of digit recognition accuracy following sudden cochlear degradation with no time to adapt. Individuals with hearing loss undergo some implicit auditory training when they ask for clarification on misheard words, therefore we are treating our more conservative model of auditory adaptation (constrained-adaption paradigm) as our most plausible model of speech perception for hard of hearing listeners. For NH-audiogram degradations (Figures 8B–D), the difference in 50% SNR between the the NH-model (Figure 8A, yellow) and the respective constrained-adaptation paradigms (Figures 8B–D, green) is −0.7, −5.7, and −9 dB SNR. For the flat-loss audiogram degradations, this metric is −4.8, −4.7, −11.1, and −11.2 dB SNR from the left to right panels (Figures 8E–H). For sloping-loss audiograms, there is a difference of −5.4, −5.4, −13.5, −13.1 dB SNR from the left to right panels (Figures 8I–L, row 3). Neither adaptation models recovered back to the level of the NH-model (Figure 8A, yellow). Both adaptation paradigms (green and red-orange) outperformed the NH-control paradigm for every cochlear state that was tested. Both adaptation paradigms recovered more task accuracy at higher SNRs than at lower SNRs.

To quantify whether the observed differences in digit recognition accuracy among various factors were significant, we estimated the SNR corresponding to 50% accuracy for each of the 10 cross-validation folds. We performed a four-way repeated-measures ANOVA (audiometric loss by ANF loss by MOCR by adaptation) with cross-validation folds modeled as a random factor. We found a main effect on digit recognition accuracy for audiometric loss [*F*_(2, 18)_ = 293, *p* < 0.0001], ANF fiber loss [*F*_(2, 9)_ = 405.5, *p* < 0.0001], and the amount of DNN adaptation [*F*_(2, 18)_ = 347.7, *p* < 0.0001] but not for MOCR loss [*F*_(1, 9)_ = 0.47, *p* = 0.8]. Even though there was no MOCR main effect, there was a significant interaction between the ANF loss and audiometric loss [*F*_(2, 18)_ = 56.8, *p* < 0.0001]. Audiometric and the ANF loss also interacted [*F*_(2, 18)_ = 11.7, *p* < 0.001], suggesting a differential effect on the contribution of traditional and hidden hearing losses on speech-in-noise performance. The ANOVA analysis also concluded several other significant 3-way interactions that are not reported.

**Figure 9:**
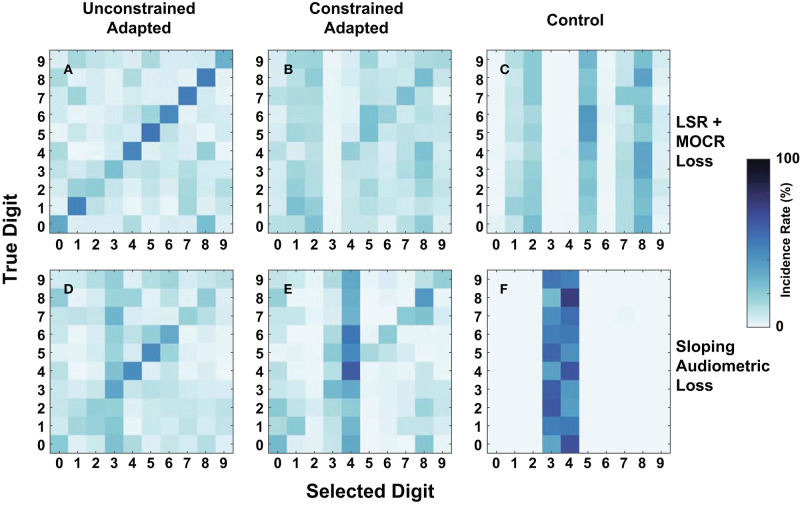
Digit-in-noise confusions for two cochlear states at −20 dB SNR. **(A–C)** Corresponds to the normal-hearing (NH) pure tone cochlear state with added medial olivocochlear reflex (MOCR) and auditory nerve fiber (ANF) dysfunction. **(D–F)** Corresponds to the sloping pure tone loss cochlear state with no added other losses. The three columns correspond to the three train/test paradigms: unconstrained-adaptation, constrained-adaptation, and NH-control (Figure 5) tested on the respective cochlear state. **(A,D,E)** Represent the confusions associated with models that produce approximately the same digit recognition accuracy. Therefore, both the cochlear state and train/test paradigm together impact how the network classifies digits.

As an additional metric of evaluation, we created confusion matrices on a subset of the cochlear-degraded speech perception models. We chose the two cochlear states situated at the extremes of the axes of degradation as case studies for the confusions in Figure 9. The first cochlear state in Figure 8I is representative of the worst hair cell degradation, which manifested as sloping audiometric threshold shift. The second cochlear state in Figure 8D reflects the cochlear state that has ANF and MOCR degradations but no pure-tone threshold degradation. Each row in Figure 9 corresponds to one of the selected cochlear states and each column represents one of three train/test paradigms.

Figures 9A,D show that of the three paradigms evaluated, the unconstrained-adaptation paradigm for both of the cochlear states most closely resembles the confusion symmetry across the diagonal seen in Figure 7's human confusions. Figures 9A,D have an RMSE of 12.2 and 17.8 relative to Figure 7A. Figures 8D,I indicate that the unconstrained-adaptation paradigm for both cochlear states produced a 30% accuracy at −20 dB SNR. Given that digit recognition accuracy was the same, any differences seen between these confusions can be attributed to how the cochlear state impacts the neurogram and the resulting DNN. The sloping loss condition has a failure mode where the model consistently misclassifies other digits as the digit three (Figure 9D); we did not observe this phenomenon in the other condition examined (Figure 9A).

Figures 9D,E show that in the sloping audiometric loss condition, both the unconstrained-adaptation and constrained-adaptation paradigms produced ~30% accuracy at −20 dB SNR. When allowed to fully adapt, the network made confusions that had more diagonal symmetry than the constrained-adaptation paradigm. The constrained-adaptation paradigm produced confusions that have a more enhanced non-uniform failure mode in comparison to the unconstrained-adaptation paradigm (Figure 9E).

### 3.3. Characterizing Neural Adaptation in DNN Following Cochlear Degradation

#### 3.3.1. Model Accuracy

**Figure 10:**
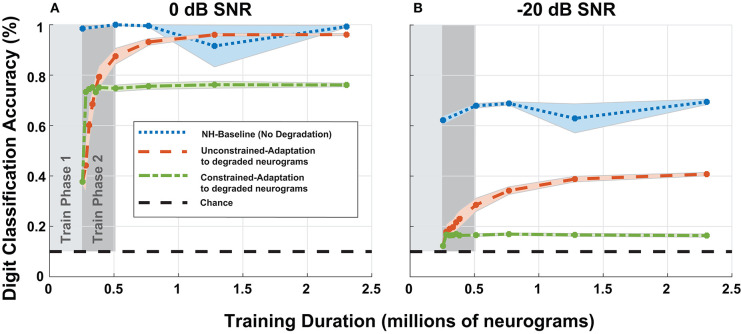
Digit recognition accuracy recovery as a function of training on degraded neurograms. **(A,B)** Contains accuracy recovery at 0 and −20 dB SNR. The blue data series shows the model accuracy achieved when we trained and tested the DNN on normal-hearing (NH) data. This data series serves as the upper accuracy bound at each sampled training duration point. After training on 250,000 NH neurograms, we trained the two adaptation networks on degraded neurograms to simulate adaptation to the acquired hearing loss. The two adaptation models both show rapid growth in digit recognition accuracy starting at the end of the first phase and behave differently as training continues (red-orange and green).

To study adaptation to hearing loss, we analyzed the digit recognition accuracy of the digit classification network as a function of training iterations. Previously, in Figure 8, we fixed the duration of the second phase of training, but for Figure 10, we extended the training duration of the second phase. We chose to focus on the most significant cochlear degradation state (i.e., sloping audiometric loss compounded with MOCR and ANF losses) since this condition likely requires the most adaptation. Figures 10A,B shows model accuracy at 0 dB SNR as a function of training duration. Figure 10 contains accuracy values beginning at the end of the first training phase on normal-hearing neurograms.

The NH-baseline network obtained 40% accuracy on degraded neurograms at the end of phase 1 of training. However once exposed to degraded neurograms during training, the adaptation models exponentially improved during training phase 2 (shown in dark gray). The constrained-adaptation paradigm (green) plateaued soon after exposure to degraded neurograms, while the unconstrained-adaptation paradigm (red-orange) continued to improve its digit recognition accuracy over a longer training duration. After training on ~1 million neurograms, at 0 dB SNR the model recovered to NH accuracy, but not at −20 dB SNR, illustrating non-linear adaptation over SNR. The constrained-adaptation paradigm, which simulates a less plastic central auditory system, never fully adapted at either SNR.

#### 3.3.2. Model Parameters

**Figure 11:**
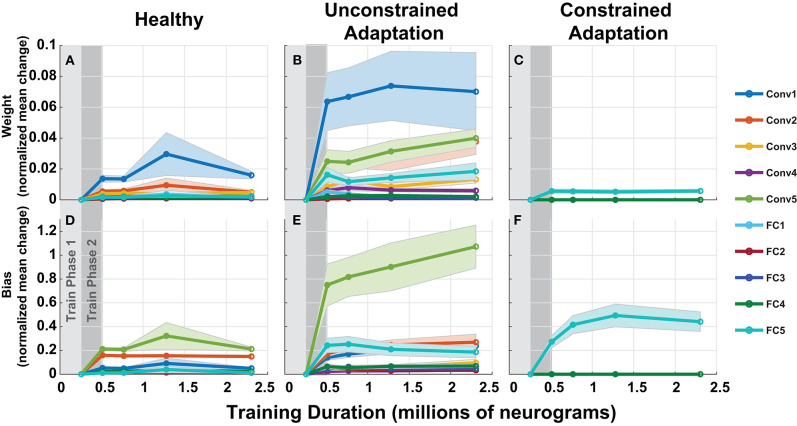
Adaptation model parameters vs. training iteration. Each panel contains a metric of normalized mean network difference relative to the normal-hearing (NH)-baseline. **(A–C)** shows normalized mean difference for each of the 10 layers' weights, while **(D–F)** shows the normalized mean difference for the 10 layers' bias. The columns correspond to the NH-control, unconstrained-adaptation, and constrained-adaptation train/test paradigms.

To understand how the networks from Figure 10 adapts to hearing loss, we inspected the parameters over each of the layers of the network in Figure 11. Each column in Figure 11 represents one of the three paradigms used in Figure 10. Figure 11 contains the normalized mean parameter change for each network layer. Each data series is constructed of the mean and standard error of the metric computed across 10 folds. For each of the twenty layers in our network, we compute the absolute mean scaled difference for each of the n parameter values in the layer's multi-dimensional parameter vector, *P*. The difference is computed against the corresponding layer's baseline mean, μ_*B*_ and is scaled by the corresponding layer's baseline standard deviation, σ_*B*_. This metric is defined in Equation (3) below:

(3) $$\begin{array}{r} \text{Normalized Mean Difference}=\frac{1}{n}\overset{n}{\underset{i=1}{\sum }}\left|\frac{\left(P_{i}-\mu_{B}\right)}{\sigma_{B}}\right|. \end{array}$$

As expected, the constrained-adaptation paradigm showed no changes except in the very last layer (Figures 11C,F). Figure 11A illustrates that convolutional weights, in addition to the final fully connected layer weights, are the most susceptible to unconstrained adaptation. When left unconstrained, the training algorithm alters the first convolutional layer the most, instead of the last fully connected layer. This indicates that changes in the first stages of processing following the auditory nerve appear to be advantageous for behavioral performance prediction in this model.

## 4. Discussion

We created a two-stage model to simulate human digit-in-noise speech recognition performance under several types of dysfunction applied to the cochlear periphery. The model achieved sigmoidal, human-like performance across SNRs with normal-hearing cochlear settings and 50% digit recognition accuracy at an SNR of −22 dB. These model results are comparable to the eight NH participants who achieved 50% digit recognition accuracy at −21 dB SNR on the same task. At this NH-audiogram setting, simulated ANF loss produced NH-like performance at high SNRs, but an ~20% loss in digit recognition accuracy at low SNRs. This SNR-dependent digit recognition accuracy loss is not a weakness of the model. Rather it is actually evidence of our model behaving like listeners who are described to have healthy audiograms but have trouble discerning speech in noisy settings.

### 4.1. Evaluation of Normal-Hearing Model

Figure 6 shows relatively well-matched, sigmoidal-shaped speech-in-noise performance between audiometrically normal-hearing listeners and our computational model. The man-machine gap is especially small when we use the commonly-used metric: the SNR at which 50% accuracy is achieved. We did not constrain the DNN to produce a sigmoidal-shaped performance, it performed as so on its own. We used the conventional cross-entropy loss function that weighs training samples equally across SNR. Our model shows a 1.3dB SNR man-machine gap that is comparable to other computational models that perform in stationary noise (Schädler et al., 2016). However, because we are reporting the complete simulated psychometric functions, we see that at higher SNRs, the model does not meet human performance perfectly. The model performance values are remapped to the human performance space so the focus is on the relative model digit recognition accuracy differences across train/test paradigms and cochlear degradations. There are a few reasons why our model performance might be slightly less than optimal. Primarily, the source could be simplifications made at the peripheral and central processing model stages may be the source. One explanation is that we removed the fine structure of speech when we smoothed the cochlear model spike response with an 8 ms time window similar to (Zilany and Bruce, 2007; Bruce et al., 2015) at each CF. Fine structure is known to be important to both pitch and speech perception, especially in fluctuating background noise (Moore, 2008). In the present study, we used a stationary white noise background, so the effect may be less pronounced than for other noise types. Our method smooths the spiking behavior at each frequency channel, which primarily captures the envelope of the neural signal, in order to dramatically reduce the computational complexity of training the DNN. A second potential source of simplification is our use of a relatively common network structure without significant architecture variation exploration. A hyperparameter search over the many possible DNN architectures may have closed the digit recognition accuracy at high SNRs. Since the system's digit recognition accuracy drops off and reaches chance at a similar SNR to our behavioral results (−30 dB), we may reject that the model over-fit. Additionally, since we kept talker training and test separate, our test sets included only novel talkers, ensuring that the network generalizes.

### 4.2. Evaluation of Cochlear Degradation Models

A major contribution of this paper shows how unique combinations of peripheral dysfunction can impact simulated digit-in-noise accuracy. Our results suggest the overall simulated performance (i.e., digit recognition accuracy or % threshold in dB SNR) can be influenced by several different types of peripheral dysfunction. This illustrates a model-based explanation for why the diagnosis of ‘hidden hearing loss’ may be so challenging (Bramhall et al., 2019). Our ANOVA analysis determined a main effect on performance for two of three of our cochlear degradations – ANF loss and audiometric loss. Our model shows a relationship between ANF loss and simulated performance which demonstrates that such “hidden” cochlear degradations could likely impact on speech perception. The audiometric loss also had an impact on performance. We were not expecting this factor given the clinical disconnect seen between pure-tone audiometric threshold and speech perception ability. Central factors could also have an impact on the diagnosis and these include overall executive function and an individual's ability to adapt following a hearing loss. Many of the simulated cochlear degradations cannot be easily validated using behavioral testing since there is no accepted non-invasive clinical measure for MOCR and ANF dysfunction. We did however find that loss of the low and medium spontaneous rate fibers in our cochlear model reduced both subjective and objective quality of the neurograms in Figure 4, and the resulting performance on a digit-in-noise task in Figure 8. This finding is consistent with hypotheses of cochlear synaptopathy impacting communication in noise (Kujawa and Liberman, 2009; Liberman et al., 2016). Our results show that complete elimination of low and medium spontaneous rate ANFs, in addition to a 14% loss in high spontaneous rate ANFs, does have an impact on simulated speech perception. It would be interesting to use the model to study simulated speech perception given severe high spontaneous rate ANF loss in order to compare the two competing theories of ANF degradation's impact on speech perception (Furman et al., 2013; Carney, 2018).

To put our NH-model and three paradigms' cochlear-degradation-dependent task accuracies into context, we looked to published behavioral speech perception psychometric curves taken from NH listeners and listeners with various audiometric profiles. We then compared the relative difference in 50% SNR between the NH-model and the NH-control paradigm tested on the 11 other cochlear degradations. Both Pichora-Fuller et al. (1995) and Bernstein and Grant (2009) recruited NH listeners and listeners that had high-frequency sloping hearing loss (similar to our modeled audiometric profile) and ran speech perception testing in the presence of stationary noise. Bernstein and Grant (2009) reports an ~5 dB difference in 50% SNR between NH listeners and listeners with hearing loss. Pichora-Fuller et al. (1995) compares NH listeners, listeners with moderate hearing loss, and listeners with severe hearing loss. They found an approximate 3.1 and 6.2 dB difference in 50% SNR in their listener groups with moderate and severe hearing loss relative to their NH group. Our three audiometric profiles with various combinations of ANF and MOCR cochlear degradation from Figure 8 can be compared to these profiles. For this comparison, we used the constrained-adaptation paradigm (green) because listeners with hearing loss implicitly undergo some amount of training as a result of requesting speech clarification during conversation.

Since there is currently no way of sub-categorizing individuals with NH audiograms based on ANF and MOCR function, we compared the range of 50% SNR losses across a given audiometric profile. The constrained-adaptation paradigm tested on the four neurograms with NH-audiograms had a difference in 50% SNR relative to the NH-model between [−0.7, −9] dB SNR (Figures 8A–D). The sloping-loss audiometric-tested constrained-adaptation models had a difference in 50% SNR relative to the NH model between [−5.4, −13.1] dB SNR (Figures 8I–L). Our results suggest that a combination of loss to the MOCR and ANF synapses may be a mechanism that could explain perceived difficulties in background noise (Kujawa and Liberman, 2009; Brown et al., 2010; Clark et al., 2012; Smalt et al., 2014). Pichora-Fuller et al. (1995) reported a 3 dB and 6 dB difference in the 50% SNR between NH listeners and listeners with moderate hearing loss. This 50% SNR difference justifies that our model provides a proper order of magnitude in performance degradation for conservative cochlear degradations (Figures 8B,C,E,F,I,J). Our more degraded cochlear states (Figures 8D,G,H,K,L) showed a higher degree of performance degradations than published behavioral data, indicating that the 12 cochlear states we selected for this proof of concept work, may be too extreme to match the physiology of listeners who are hard of hearing. For example, we compared healthy vs. completely destroyed low and medium ANFs in the modeled periphery, and this may not be realistic for the human populations we aim to model. In the future, we may use more conservative amounts of degradations of the MOCR and ANFs. Given a comprehensive review of a large data base of speech perception performance as a function of SNR and audiometric profiles, we may be able to justify whether the variability along a given audiometric profile is plausible.

### 4.3. Adaptation Following Peripheral Damage

Figure 8 shows a wide range of plasticity in response to peripheral dysfunction (unconstrained and constrained adaptation paradigms). For NH pure-tone audiogram simulation with MOCR loss, both models with adaptation perfectly recovered the NH-model accuracy across all SNRs (Figure 8B). For a majority of the other combinations of audiometric, ANF, and MOCR degradations (Figures 8C–J), both adaptation models recovered accuracy at high SNRs but not at low SNRs. Our ANOVA analysis also determined a main effect of test/train paradigm (i.e., amount of adaptation) on accuracy. Figure 10 illustrates that with more training, even the most degraded cochlear case (Figure 8L), recovers the 0 dB SNR accuracy achieved by the NH-model. Although there is no human ground truth for our exact two adaptation models, the constrained-adaptation model accuracy seen in Figure 8 is similar in nature to that seen in Whitton et al. (2017). Whitton et al. (2017) performed a behavioral study which quantified the impact of 8 weeks of closed-loop audiomotor training on a speech perception task in hearing aid users. Even after training, their subjects did not recover 100% accuracy at all SNRs; they demonstrated more improvement at higher SNRs than at lower SNRs. The preservation of the sigmoidal accuracy throughout our static and plastic models is a strength of the model. Our model may be successfully simulating the information loss in the periphery, enough to counteract the propensity for a DNN to tune its parameters to create an optimal, flat accuracy over SNR. Whitton et al. (2017)'s task accuracy gains are more conservative than the accuracy gains attained by our adaptation models. This may be because of the extreme cochlear degradations and plasticity we chose to model. In the next iteration of the model, more conservative cochlear degradation states and degrees of plasticity will be used. Another way to validate the model would be an animal study that attempts to quantify how much adaptation to a stimuli discrimination task can result from intensive training following controlled cochlear degradation. Such an experiment like this would help determine whether plasticity is degradation dependent. Although the accuracy gains achieved by our models of adaptation may not have a behavioral ground truth, the analysis done on the networks does provide a framework for studying models of adaptation in the future.

Figures 10, 11 illustrates how fast a network learns and where the network changes as it adapts to new stimulus input properties. Continued training of our model after peripheral loss resulted in further accuracy gains, as shown in Figure 10. Often times continued training can lead to over-fitting and accuracy can even drop or oscillate. It is an open question as to how much adaptation can occur to the neural substrate of the auditory pathway in humans, but it is generally thought that more peripheral regions are less plastic (Irvine and Rajan, 1995). We attempted to model central plasticity by fixing all the layers of the DNN except the final layer because the later fully connected layers could be interpreted as the task specific decision making portion of the cortex of our network. When we analyzed the location of network adaption, we discovered that when left unconstrained, the majority of relative weight changes relative occur at the first input convolutional layer, where adaptation in humans may not be possible (Figure 11).

### 4.4. Future Work

Several improvements and extensions could be made to our model architecture. It is a possibility that the dimensionality reduction that is currently required to keep processing time manageable did in fact prove detrimental to the NH-model accuracy. Given this constraint, there is potentially a better use of the neurogram dimensions that would keep computation the same and potentially increase accuracy. It may be fruitful to redistribute the dimensionality of the neurogram array such that the frequency and time axes could be adjusted by an equal factor. This would provide the capacity to have a higher resolved time dimension to capture short duration speech events like consonants, while keeping a competitive frequency resolution in the ASR space (Schilling et al., 2020). Additionally, the classifier could be extended to recognize a larger vocabulary set, as done in Kell et al. (2018). With regard to the neural input, exploring the effects of the fine structure and envelope representations of speech on classification could reveal their relative importance to the type of hearing loss. In this work, we did not study the effect of other types of background noise (such as multi-talker babble) and the effects of reverberation. These conditions could be particularly important for studying the effects of cochlear damage.

One future application of this model is for system identification, iteratively adapting peripheral model parameters, such as MOCR and ANF, to match overall human performance or word confusions for individual subjects. If an individualized model of the cochlear pathway can predict the confusion patterns of a listener, it could guide rehabilitation or treatment strategies. One of the primary reasons for extending both the peripheral fidelity and noise environment realism for this model is for acoustic enhancement. Our view is that an end-to-end system of speech classification, such as the one developed here, could be pre-pended with an enhancement system that could be interactively trained. Our model would act as a surrogate for the human, and could be trained on many possible enhancement algorithms and hyper-parameters. To improve computational run time, the periphery itself could perhaps be replaced by a neural-network based cochlear model (Baby et al., 2020), although the ability to simulate individualized cochlear settings once the model is trained may be challenging. It is because of this fact that hybrid phenomenological-neural-network models may hold promise for studying the peripheral and central nervous system. With regards to rehabilitation and enhancement, the more a surrogate model could match any peripheral dysfunction, the more likely the enhancement would be to succeed.

## 5. Conclusion

Our goal in this paper was to develop a model of speech perception with underlying cochlear functionality to simulate the impact of various types of peripheral hearing loss on simulated speech-in-noise performance. We found that our DNN-based approach for accomplishing a digit-in-noise task was able to replicate human performance as compared to normal-hearing approximately listeners. A sudden hearing loss introduced into the model, followed by a conservative amount of training, produced simulated performance that was consistent with published behavioral speech-in-noise testing.

Future application of this model could serve to translate results discovered in animal studies into evidence for or against hypothesized sources of speech-in-noise difficulties. Ultimately, such a model could be matched to individual behavioral responses and speech perception performance. This would allow for the optimization of rehabilitation strategies including targeted acoustic enhancement, as well as for deriving treatments and strategies for optimizing adaption to hearing loss.

## Data Availability Statement

The datasets analyzed for this study can be found in the Linguistic Data Consortium https://catalog.ldc.upenn.edu/LDC93S10.

## Ethics Statement

The studies involving human participants were reviewed and approved by Massachusetts Institute of Technology (MIT) Committee on the Use of Humans as Experimental Subjects (COUHES). The participants provided their written informed consent to participate in this study.

## Author Contributions

SH: formal analysis, DNN architecture, software development, visualization, writing-original draft preparation, and writing-review and editing. CS: conceptualization, formal analysis, funding acquisition, methodology, project administration, software, supervision, validation, writing-original draft preparation, and writing-review and editing. GC: algorithm development, DNN architecture, software, validation, and writing-review and editing. TQ: conceptualization, writing-review, editing, and faculty advisor. All authors contributed to the article and approved the submitted version.

## Conflict of Interest

The authors declare that the research was conducted in the absence of any commercial or financial relationships that could be construed as a potential conflict of interest.
